# Medical device regulation (MDR) in health technology enterprises – perspectives of managers and regulatory professionals

**DOI:** 10.1186/s12913-023-09316-8

**Published:** 2023-03-30

**Authors:** Juhamatti Huusko, Ulla-Mari Kinnunen, Kaija Saranto

**Affiliations:** Department of Health and Social Management, University of Eastern Finland, Yliopistonranta 1, Kuopio, 70210 Finland

**Keywords:** Health Technology, Medical device, Regulation, Health Care, Information needs, Information seeking, Information use

## Abstract

**Background:**

In the European Union (EU), there are over half a million medical devices, varying from pacemakers to software. Medical devices play an important role in health care as they are used in diagnosis, prevention, monitoring, prediction, prognosis, treatment, or to alleviate disease. Medical devices are regulated in the EU by the Medical Device Regulation (MDR), which came into force on 25 April 2017 and into application on 26 May 2021. The demand for regulation arose from the need to establish a transparent, robust, predictable, and sustainable regulatory framework. This study aims to examine how the managers and regulatory professionals in health technology enterprises perceived the application of the MDR and what were their information needs regarding the MDR.

**Methods:**

A link to an online questionnaire was sent to 405 managers and regulatory professionals representing health technology enterprises in Finland. The study included 74 respondents. Descriptive statistics were used to describe and summarise the characteristics of the dataset.

**Results:**

Information related to the MDR was fragmented and the necessary information was sought from multiple information sources, while the Finnish Medicines Agency (Fimea) was regarded as the most important source of information and training provider. To some extent, the managers and regulatory professionals expressed their dissatisfaction with the performance of Fimea. The managers and regulatory professionals were not very familiar with the ICT systems provided by the EU. The size of an enterprise affected how many medical devices it manufactures and generally affected the views about the MDR.

**Conclusions:**

The managers and regulatory professionals understood the role of the MDR regarding the safety and transparency of medical devices. The available information regarding the MDR did not properly fit the needs of users and there seemed to be a gap in information quality. The managers and regulatory professionals had some difficulties understanding the available information. Based on our findings, we believe it is paramount to evaluate the challenges faced by Fimea and how it could improve its performance. To some extent, the MDR is regarded as a burden for smaller enterprises. It is important to highlight the benefits of ICT systems and to develop them to better meet the information needs of enterprises.

## Introduction

The European Union (EU) has an innovative medical device sector in which small and medium-sized enterprises (SMEs) are at the forefront. The regulatory framework for medical devices enables a well-functioning European single market. The medical device regulations aim to ensure a high level of health protection for patients and users [1]. The Medical Device Regulation, MDR 2017/745 (hereinafter referred to as “the MDR”) came into force on 25 April 2017 and into application on 26 May 2021. The MDR replaced the Medical Device Directive, MDD 93/42/EEC, as well as the Council Directive 90/385/EEC on Active Implantable Medical Devices, AIMD [2]. All medical device manufacturers are obligated to comply with the requirements of the MDR, including the quality management system and market surveillance [3]. In April 2020, the European Commission announced that, due to the COVID-19 situation, it had postponed the transition period of the MDR by one year from May 2020 to May 2021. The decision was influenced by the challenges posed by COVID-19 and the need to increase the availability of medical devices. The aim of postponing was to ensure patient safety and reduce the pressure on authorities, enterprises and Notified Bodies (NBs) [4]. In practice, the transition to the MDR means that, as of 26 May 2021, it is not possible to introduce new medical devices on the EU market that do not meet the requirements of the MDR.

The medical device sector is important for the European economy and there are over 500,000 medical devices on the EU market [1]. It is estimated that the global market has a total of two million medical devices and the devices are categorised into around 7000 device groups [5]. These medical devices vary from contact lenses to pacemakers and software. Medical devices play an important role in supporting patients and healthcare providers by offering innovative solutions for diagnosis, prevention, monitoring, prediction, prognosis, treatment, or to alleviate disease [1].

The demand for new regulation arose from the need to establish a transparent, robust, predictable, and sustainable regulatory framework, [3] which aims to (1) ensure high-level protection of human health and safety, (2) ensure a functional internal market and (3) provide a regulatory framework, which supports innovation and the competitiveness of the medical device industry in the EU [6]. The previous directives had weaknesses which emerged, for example, in the breast and hip implant scandals [7]. A breast implant scandal occurred a decade ago when a French enterprise called Poly Implant Prothèse was caught using industrial-grade silicone instead of medical-grade silicone in its breast implants. The implants had serious safety issues since they had a higher risk of rupturing [8, 9]. However, the Scientific Committee on Emerging and Newly-Identified Health Risks (SCENIHR) did not find evidence that implants are associated with breast cancer or anaplastic large cell lymphoma [10]. The hip implant scandal happened in 2010 following a recall of ASR (Articular surface replacement) hip implants made by DePuy, a subsidiary of Johnson and Johnson [11]. These metal-on-metal (MoM) hip implants were withdrawn from the markets due to the high revision rates [12]. ASR implants had caused considerable pain and disability in patients due to metal debris that destroyed the soft tissues surrounding the joint [11].

Each EU country has its national authority that supervises the regulatory compliance of medical devices and operators [13]. Fimea supervises the pharmaceutical sector and the regulatory compliance of medical devices and operators in Finland. Fimea is a central administrative agency that operates under the Ministry of Social Affairs and Health. Supervision is conducted in collaboration with other EU authorities [14]. In Finland, the National Medical Devices Act 719/2021 supplements the EU regulations on medical devices, such as the MDR [15].

### The MDR and the challenges

The MDR divides medical devices into four main categories: Class I, IIa, IIb and III. In addition, devices in Class I have three subclasses: Is, sterile condition, Im, measuring function and Ir, reusable surgical instruments. A Class III device is considered a high-risk device and therefore has the most stringent requirements [3, 16]. In practice, the MDR impacts the classification of some devices and, consequently, certain medical devices will be moved to a higher class. The MDR aims to bring more effective and safer medical devices to the markets [17]. The MDR sets new standards for clinical trials since the data must be robust and reliable. The quality and safety requirements for the performance of clinical trials are similar to studies conducted in the pharmaceutical industry [18].

Software plays a significant role in modern medicine, and although the software was already included in previous directives, its description has been detailed in the MDR [19]. The MDR contains precise details for the requirements of software qualification and classification, which have been highlighted particularly in the classification rule 11 of the MDR [20]. MDR plays an important role in ensuring the safety of medical device users and should not be seen as an obstacle to the birth of healthcare innovations, but as an enabler [19].

When discussing the improvements to the MDR, it is important to clarify essential ICT systems. The NANDO database – New Approach Notified and Designated Organisations – offers information about Notified Bodies which, in this context, are organisations that assess the conformity of medical devices before they enter the market. Notified Bodies are designated by an EU country [21]. According to NANDO, there are two designated Notified Bodies in Finland regarding the MDR: SGS Fimko Oy (NB 0598) and Eurofins Expert Services Oy (NB 0537) [22]. The EUDAMED database – European Database on Medical Devices – provides information about the lifecycle of medical devices available in the EU. The database has been developed by the European Commission and contains information about economic operators such as manufacturers, authorised representatives and importers [23].

It is important to note that medical device regulations and standards evolve slowly, and it might be difficult to apply them to the latest technology. It could be that regulations and standards are outdated or unsuitable when medical devices are used outside of clinical settings, for example, in patients’ homes. In some cases, regulations can inhibit innovations if a new technology is not directly beneficial to the healthcare system due to time or cost constraints. Also, in some cases, existing systems cannot be adapted to new technology [24]. As the complexity of health care increases with the range of diagnosis and treatment options, it is important to carefully evaluate whether the use of new technologies is safe for patients. New technologies such as artificial intelligence (AI) can contribute to patient safety, but they can also compromise it [25].

In the EU, medical devices require a CE marking, which proves that the product meets the essential requirements and has been evaluated under the requirements. The product’s manufacturer or authorized representative declares that the product meets the standards regarding safety, health and environmental protection and consumer protection [26]. Obtaining CE marking is a real challenge for the competitiveness of medical device manufacturers since the majority of them are SMEs [27]. According to a recent study, the MDR will increase patient safety but as a downside, the increased costs and administrative burden of MDR will have an impact on the innovativeness of the SMEs. As a result, some of the enterprises decide to focus on “non-medical” products [28]. The regulation of medical devices is less developed than the regulation of medicinal products. However, the current medical device classifications are complex and designed primarily for regulators. A simpler classification has been proposed based on the application site of the device, the time scale of use, and whether the device has an external power source. This proposed classification could be used to clarify which devices would be classified as medicines and which as medical devices. For example, medical devices designed for dispensing drugs, such as prefilled syringes, must meet the requirements set for medicinal products [29]. There have also been criticisms towards conformity assessment of medical devices. In the EU, conformity assessment has been delegated to private companies, known as Notified Bodies and it has been perceived as problematic. Notified Bodies operate in competitive markets, competing against each other and adopting market behaviour, which is not necessarily ideal when considering their role in public health [30].

### The Finnish healthcare system context

In Finland, a health and social services reform is underway. In future, the wellbeing services counties will be mainly responsible for health, social and rescue services instead of the municipalities and hospital districts, which are currently responsible for organising such services. This reform is necessary since the population in Finland is ageing and there is an increasing need for care services. Also, the birth rate is falling, the working-age population is declining, and tax revenues are therefore decreasing. In the reform, the public sector will become the primary health and social services provider and the private sector will act as a supplementary service provider. The reform aims to integrate health and social services and it will shift the focus to resolving health problems at an early stage and speeding up access to treatment [31]. The reform is also needed from an economic point of view. In Western countries, healthcare costs are rising as populations age and the demand for health services increases. The costs of health care are rising in almost all countries [32 (p.14)].

In 2015, the Ministry of Social Affairs and Health published a strategy called *Information to support well-being and service renewal - eHealth and eSocial Strategy 2020*, which supports the improvement of information management and digital services in health and social care reform. The strategy aims to secure the flow of information and make healthcare services more customer orientated. The strategy states that Finland is actively participating in strategic and operational cooperation at the EU level and is seeking to proactively influence the development of EU regulations concerning health care. The strategy also emphasises that health technology is evolving at a rapid pace, and it is important to use health technology in a controlled way in health and social care. Collaboration between technology developers and the health and social care sector benefits both parties [33].

In this study, medical devices that fall under the MDR are regarded as health technology devices. The importance of health technology devices and health data in the care of patients is constantly growing. Health technology plays a key role in almost every patient encounter, and, for example, modern diagnostics is challenging without health technology [32 (p.25)]. Health technology is a significant industry for the Finnish economy; its exports in the 2021 totalled EUR 2.52 billion [34]. Overall, health technology is an essential part of Finnish healthcare, as the ageing population will increase the demand for health technology [35 (p.5)]. Healthcare in Finland is highly digitalized among the public and private sector service providers, which offer a wide range of e-health services for citizens [36].

The COVID-19 pandemic expanded the capacity of health institutions and health systems all over the world and accelerated the adoption of new technologies such as digital health technology [37]. During the pandemic, digital health technologies were used for patient screening and treatment as well as to support healthcare providers [38]. The use of health technology often changes treatment processes, forcing health professionals to adapt to new practices. In addition, technological solutions need to be adaptable to different care environments and, for representatives of health technology enterprises, it may be challenging to understand the boundaries of medical professionalism [32 (p.66)]. In the next section, we will describe the objectives of this research.

## Objectives

This study aims to examine how managers and regulatory professionals in health technology enterprises perceive the application of the MDR and what are their information needs regarding the MDR.

This study posed the following questions:

1. How do managers and regulatory professionals in health technology enterprises perceive the application of the Medical Device Regulation (MDR)?
2. What are the information needs of managers and regulatory professionals in health technology enterprises regarding the Medical Device Regulation (MDR)?

## Methods

The target group enterprises in the health technology sector represent different industries based on their focus. The total number of health technology enterprises varies since, in a modern economy, the number of enterprises is constantly changing. Especially, the identification of health technology enterprises in Finland is challenging, because there is no health technology industry classification in the Standard Industry Classification known as TOL (Toimialaluokitus) 2008 [39].

In this study, a list of recipients was created by using the member lists on the websites of health technology associations Healthtech Finland [40] and Sailab [41] and the EUDAMED [23] database. At the time of the study, the EUDAMED database contained the contact information for 214 medical device manufacturers registered in Finland [23]. Also, more recipients were found on the websites of Terkko Health Hub [42] and Health Incubator Helsinki [43]. The list of recipients was completed manually by searching for potential respondents via Google searches. An email list comprising 405 recipients was created.

### Data collection

A questionnaire was chosen as a data collecting method since it offers a way to obtain information from a large group of people. Questionnaires also maintain respondents’ anonymity and they lack interviewer bias [44]. The questionnaire was primarily based on a previous questionnaire from a study that focused on the transitional period of MDR among the Finnish health technology enterprises [45]. Minor modifications were made to the questionnaire to make it more suitable for data collection after the application of the MDR. The questions were based on the MDR and were supplemented with discussions with medical device consultants and representatives of health technology enterprises. In this context, medical device consultants refer to people who advise health technology enterprises on regulatory matters.

The Finnish-language online questionnaire was created using the Webropol online survey tool. The questionnaire comprised 26 single and multiple-choice questions, dropdowns, and questions asking the respondents’ opinions about the MDR by giving a quantitative value using a five-point Likert scale: strongly agree, agree, neither agree nor disagree, disagree, and strongly disagree. The questionnaire also included six optional open-ended questions. These questions enabled the respondents to clarify, elaborate on certain statements and give feedback about the questionnaire. The background variables were as follows: location of the enterprise, enterprise size, turnover, year of establishment, sex, age, education level and company position. The questionnaire was pretested by a total of four people: a health technology entrepreneur, a health technology business consultant, a legal expert and a financial expert. The health technology entrepreneur suggested that minor improvements could be made to the phrasing. Based on these suggestions, changes were made, and it was determined that the questionnaire worked properly.

The data for this anonymous study were collected from the representatives of health technology enterprises in Finland between February and March 2022. The data collection process was two-fold. The data were primarily collected via the questionnaire, which was sent directly to 405 recipients. Two reminders were sent to the recipients. Secondly, the data were collected by sharing the questionnaire through LinkedIn and distributing it in Healthtech Finland’s and Helsinki Health Capitals’ newsletters. The aim of this two-fold data collection process was not to compare the data but to reach as many respondents as possible. 66 out of 405 recipients responded to the questionnaire, generating a 16.3% response rate. In addition, eight respondents from LinkedIn and the newsletters of Healthtech Finland and Helsinki Health Capital completed the questionnaire. A total of 74 responded to the questionnaire.

### Data analysis

First, the data (n = 74) from two datasets were combined into one dataset, which was analysed using IBM SPSS Statistics 28 statistical software. Due to the relatively small dataset, the data were analysed using descriptive statistics to provide frequencies, means and percentages, and by performing cross-tabulation. In current organizational research, complex analyses are popular and only scant attention is paid to descriptive statistics. The challenge with the complex and diverse data-analytic methods is that they have been (a) applied and interpreted incorrectly, (b) dependence on significance testing has increased and (c) interpretation has become more difficult, resulting in a gap between science and practice. More simpler and informative presentations of descriptive statistics would increase the value and interpretability of the research [46]. The five-point Likert statements *strongly agree, agree, neither agree nor disagree, disagree, and strongly disagree* were recoded into three variables: *agree, neither agree nor disagree, and disagree.* The background variable describing an enterprise’s size was recoded into four categories depending on the number of employees: micro (1–9 employees), small (10–49 employees), medium-sized (50–249 employees) and large (250 + employees) enterprises. This was in order to classify the enterprises according to their size in the classifications used in the EU [47]. The open-ended questions were not analysed in this study. The results are presented in the tables.

### Ethical considerations

This study follows the guidelines of the Finnish National Board on Research Integrity, whose task is to promote good scientific practice in the Finnish scientific community [48] In addition, this study follows the good research practices of the All European Academies (ALLEA), which are reliability, honesty, respect and accountability [49]. The acquisition of research data did not require permission from the enterprises. The first question in the questionnaire ensured that the respondents represented a health technology enterprise that manufactures or is developing medical device(s) that fall under the MDR. The second question asked the respondents for their informed consent to participate in this study. If a respondent answered “No” to either question, they could no longer take part in the study.

## Results

Almost half (45.9%, n = 34) of the enterprises were micro enterprises, 35.1% (n = 26) small enterprises, 10.8% (n = 8) medium-sized enterprises and 8.1% (n = 6) large enterprises. Most of the respondents were academically qualified as 62.2% (n = 46) of them had a graduate degree and 13.5% (n = 10) a doctoral degree. Around one third (35.1%, n = 26) of the respondents were either CEOs or quality managers/directors (Table 1**).** A total of 75.6% (n = 56) of the enterprises were established in the 2000s while the oldest enterprise was established in 1881. The average age of the respondents was 46.82 years, the youngest respondent was 24 years old and the oldest was 66 years old.

**Table 1:** Respondent profiles, n = 74

Enterprise size	%	n
Micro (1–9 employees)	45.9%	34
Small (10–49 employees)	35.1%	26
Medium-sized (50–249 employees)	10.8%	8
Large (250 + employees)	8.1%	6
**Sex**		
Male	58.1%	43
Female	36.5%	27
Prefer not to answer	5.4%	4
**Education level**		
Comprehensive school (lower secondary) (EQF* Level 2)	1.4%	1
Upper secondary education or vocational education (EQF Level 4)	2.7%	2
Undergraduate degree (EQF Level 6)	20.3%	15
Graduate degree (EQF Level 7)	62.2%	46
Doctoral degree (EQF Level 8)	13.5%	10
**Company position**		
Chief executive officer (CEO)	35.1%	26
Chief technical officer (CTO)	9.5%	7
Quality manager or director	35.1%	26
Chair of the board (COB)	6.8%	5
Other	25.7%	19

*EQF = European Qualifications Framework.

Note: Percentages may not total 100% due to rounding or multiple responses.

Most (81.1%, n = 60) of the respondents stated that their enterprise had a designated person responsible for regulatory compliance. Less than half (40.5%, n = 30) of the enterprises manufactured one medical device while 23% (n = 17) of the enterprises manufactured five or more medical devices. Software and devices (hardware) were the most common (50%, n = 37) types of medical devices. A total of 67 respondents stated they manufactured medical devices that fell under the MDR and 39 of the respondents stated their medical devices fell under the MDD. Under the MDR, around one third (36.5%, n = 27) of the enterprises manufactured Class I devices and around one third (36.5%, n = 27) manufactured Class IIa devices. Class I (25.7%, n = 19) devices were the most commonly manufactured devices under the MDD (Table 2).

**Table 2:** Enterprise profiles, n = 74

The person responsible for regulatory compliance	%	n
A person responsible for regulatory compliance in their enterprise	81.1%	60
A person permanently and continuously at their disposal	18.9%	14
**Number of approved medical devices**		
No devices approved	13.5%	10
One	40.5%	30
Two	9.5%	7
Three	8.1%	6
Four	5.4%	4
Five or more	23%	17
**Type of medical device**		
Software	50%	37
Devices	50%	37
Materials	6.8%	5
Instruments	16.2%	12
**Classification of medical devices**		
Class I (MDR*)	36.5%	27
Class Is (MDR)	2.7%	2
Class Im (MDR)	1.4%	1
Class Ir (MDR)	0%	0
Class IIa (MDR)	36.5%	27
Class IIb (MDR)	10.8%	8
Class III (MDR)	2.7%	2
Class I (MDD**)	25.7%	19
Class Is (MDD)	1.4%	1
Class Im (MDD)	1.4%	1
Class IIa (MDD)	10.8%	8
Class IIb (MDD)	9.5%	7
Class III (MDD)	4.1%	3
Our devices do not belong to the aforementioned classes	1.4%	1

*MDR = Medical Device Regulation

**MDD = Medical Device Directive

Under the MDR and MDD, Class I medical devices are low-risk devices, while Class III medical devices are high-risk devices.

Note: Percentages may not total 100% due to rounding or multiple responses.

Most (83.8%, n = 62) of the respondents regarded Fimea as being the most frequently used information source. The second most frequently (71.6%, n = 53) used source was EU websites, and medical device consultants were the third most frequently (59.5%, n = 44) used source. Multiple organisations provided training about the MDR while Fimea was the most frequently (68.9%, n = 51) used training provider. Over half (54.1%, n = 40) of the respondents participated in in-house training. Less than half (39.2%, n = 29) of the respondents had taken part in the training provided by medical device consultants. Almost three-quarters (73%, n = 54) of the respondents had participated in the training in order to understand the changes from the MDD to the MDR, and slightly over half (55.4%, n = 41) of the respondents had participated in the training in order to create a quality management system. In addition, 41.9% (n = 31) of the respondents stated that they needed training in how to develop a regulatory and registration strategy (Table 3).

**Table 3:** Information source, training provider, topic of training, n = 74

Information source for MDR	%	n
Fimea	83.8%	62
EU websites	71.6%	53
Medical device consultants	59.5%	44
Terveysteknologia ry – Healthtech Finland	33.8%	25
SGS Fimko Oy	31.1%	23
Other	24.3%	18
Sailab – MedTech Finland ry	21.6%	16
Eurofins Expert Services Oy	20.3%	15
Business Finland	18.9%	14
Foreign notified body	16.2%	12
Healthcare incubator or accelerator	10.8%	8
**Training provider regarding the MDR**		
Fimea	68.9%	51
In-house training	54.1%	40
Medical device consultants	39.2%	29
Terveysteknologia ry – Healthtech Finland	23%	17
Other	21.6%	16
Business Finland	18.9%	14
SGS Fimko Oy	17.6%	13
Sailab – MedTech Finland ry	17.6%	13
Healthcare incubator or accelerator	14.9%	11
Foreign notified body	13.5%	10
Eurofins Expert Services Oy	9.5%	7
**The topic of training regarding the MDR**		
To understand the significance of change (from MDD to MDR)	73%	54
To create a quality management system (EN ISO 13485)	55.4%	41
To develop a regulatory and registration strategy	41.9%	31
To understand the differences between different product categories (I–III)	36.5%	27
For conducting a clinical trial	32.4%	24
For conducting a usability study	21.6%	16
To understand the effect of Brexit	12.2%	9
Other	12.2%	9
We did not need training	6.8%	5
To apply for funding for the CE* marking process	4.1%	3
For EMC** testing	2.7%	2
For materials research	1.4%	1

*CE = Conformité Européenne

**EMC = Electromagnetic compatibility

Note: Percentages may not total 100% due to rounding or multiple responses.

The available information about the MDR is generally sufficient. However, 24.3% (n = 18) of the respondents did not agree when asked if the available information about the MDR was sufficient. Over half (55.4%, n = 41) of the respondents stated that there is no comprehensive website on the MDR. Less than one third (25.7%, n = 19) of the respondents stated that decision-making based on the available information was not an issue, while 59.5% (n = 44) of the respondents felt that decision-making could be quite challenging. More than half of the respondents (58.1%, n = 43) had previous experience with the CE marking process for medical devices. Also, only around one third of the respondents (35.1%, n = 26) considered that the available information was easy to understand, whereas 56.8% (n = 42) of the respondents found the available information quite hard to understand (Table 4).

**Table 4:** Adequacy of the available information and the respondent’s knowledge and expertise regarding the MDR, n = 74

Information availability of the MDR	Agree	Neither agree nor disagree	Disagree
Sufficient information available regarding the MDR*	74.3% (n = 55)	1.4% (n = 1)	24.3% (n = 18)
We know who to consult if our enterprise needs more information regarding the MDR	83.8% (n = 62)	1.4% (n = 1)	14.9% (n = 11)
Based on the available information, we can identify the impact of the MDR on our enterprise’s operations	83.8% (n = 62)	6.8% (n = 5)	9.5% (n = 7)
There is a website about the MDR that has all the information you need	27% (n = 20)	17.6% (n = 13)	55.4% (n = 41)
Decision-making based on the information available is not an issue	25.7% (n = 19)	14.9% (n = 11)	59.5% (n = 44)
**Respondents’ knowledge and expertise regarding the MDR**			
I am familiar with the MDR in general	93.2% (n = 69)	5.4% (n = 4)	1.4% (n = 1)
I know how the MDR affects the products that our enterprise makes	93.2% (n = 69)	4.1% (n = 3)	2.7% (n = 2)
I have previous experience of the CE marking process for medical devices	58.1% (n = 43)	9.5% (n = 7)	32.4% (n = 24)
I think that the available information is easy to understand	35.1% (n = 26)	8.1% (n = 6)	56.8% (n = 42)

*MDR = Medical Device Regulation.

Note: Percentages may not total 100% due to rounding or multiple responses.

Over half (55.4%, n = 41) of the respondents stated that they had received sufficient training while 25.7% (n = 19) of the respondents stated that they had not received sufficient training. Overall, the respondents found the training useful, that it provided important information and that the trainers had been sufficiently competent. A total of 73% (n = 54) of the respondents needed the support of a medical device consultant in the CE marking process. As much as 75.7% (n = 56) of the respondents felt that their enterprise had the necessary expertise for the CE marking process. Around one third (36.5%, n = 27) of the enterprises received support from another health technology enterprise. Over half (52.7%, n = 39) of the enterprises independently learned about all important aspects of the CE marking process. When looking at the reasons given for outsourcing parts of the CE marking process, the respondents stated that the most common (82.4%, n = 61) reason was a lack of knowledge and expertise in their enterprise and the second most common (74.3%, n = 55) reason was the allocation of resources (Table 5).

**Table 5:** Training, need for external support, reasons for outsourcing, n = 74

Views on training concerning the MDR*	Agree	Neither agree nor disagree	Disagree
There has been sufficient training	55.4% (n = 41)	18.9% (n = 14)	25.7% (n = 19)
The trainers were competent	74.3% (n = 55)	17.6% (n = 13)	8.1% (n = 6)
The training has provided important information about the MDR	79.7% (n = 59)	17.6% (n = 13)	2.7% (n = 2)
I found the training sessions useful	78.4% (n = 59)	16.2% (n = 12)	5.4% (n = 4)
The content of the training sessions has been suitable for our enterprise	59.5% (n = 44)	27% (n = 20)	13.5% (n = 10)
The need for external expertise in the CE** marking process			
We need the support of a medical device consultant in the CE marking process	73% (n = 54)	2.7% (n = 2)	24.3% (n = 18)
Our enterprise has the necessary expertise to carry out the CE marking process	75.7% (n = 56)	8.1% (n = 6)	16.2% (n = 12)
We receive support with the CE marking process from another health technology enterprise	36.5% (n = 27)	18.9% (n = 14)	44.6% (n = 33)
We independently learn all important aspects of the CE marking process	52.7% (n = 39)	14.9% (n = 11)	32.4% (n = 24)
**Reasons to outsource parts of the CE marking process**			
Cost savings	21.6% (n = 39)	27% (n = 20)	51.4% (n = 38)
Because of the allocation of resources	74.3% (n = 55)	10.8% (n = 8)	14.9% (n = 11)
Lack of necessary knowledge and expertise in our enterprise	82.4% (n = 61)	8.1% (n = 6)	9.5% (n = 7)
It is not possible to acquire your own expertise	31.1% (n = 23)	25.7% (n = 19)	43.2% (n = 32)
Our enterprise does not need to outsource the CE marking process	47.3% (n = 35)	27% (n = 20)	25.7% (n = 19)

*MDR = Medical Device Regulation.

**CE = Conformité Européenne.

Note: Percentages may not total 100% due to rounding or multiple responses.

When comparing the overall impact of the MDR on the enterprises, we noted that all (100%, n = 6) of the respondents from the large enterprises stated that the MDR improves product traceability while only half (50%, n = 4) of the medium-sized enterprises agreed. Similarly, all (100%, n = 6) respondents from large enterprises stated that the MDR improves patient safety. Only 38.2% (n = 13) of micro enterprises, 38.5% (n = 10) of small enterprises and 37.5% (n = 3) of medium-sized enterprises stated that the MDR guarantees fair market access, while a total of 83.3% (n = 5) of large enterprises agreed (Table 6).

**Table 6:** Overall impact of the MDR, n = 74

The MDR* has improved product traceability			Agree	Neither agree nor disagree	Disagree
Enterprise size	Micro	n	23	9	2
		%	67.6%	26.5%	5.9%
	Small	n	21	3	2
		%	80.8%	11.5%	7.7%
	Medium-sized	n	4	3	1
		%	50%	37.5%	12.5%
	Large	n	6	0	0
		%	100%	0%	0%
**The MDR has improved patient safety**					
Enterprise size	Micro	n	23	4	7
		%	67.6%	11.8%	20.6%
	Small	n	20	3	3
		%	76.9%	11.5%	11.5%
	Medium-sized	n	5	3	0
		%	62.5%	37.5%	0%
	Large	n	6	0	0
		%	100%	0%	0%
**The MDR has increased transparency**					
Enterprise size	Micro	n	21	8	5
		%	61.8%	23.5%	14.7%
	Small	n	22	1	3
		%	84.6%	3.8%	11.5%
	Medium-sized	n	3	3	2
		%	37.5%	37.5%	25%
	Large	n	5	0	1
		%	83.3%	0%	16.7%
**The MDR guarantees fair market access for enterprises**					
Enterprise size	Micro	n	13	6	15
		%	38.2%	17.6%	44.1%
	Small	n	10	5	11
		%	38.5%	19.2%	42.3%
	Medium-sized	n	3	3	2
		%	37.5%	37.5%	25%
	Large	n	5	1	0
		%	83.3%	16.7%	0%

*MDR = Medical Device Regulation.

Note: Percentages may not total 100% due to rounding or multiple responses.

The respondents were asked about the ICT systems in the MDR. Half (50%, n = 17) of the micro enterprises, 53.8% (n = 14) of the small enterprises, 37.5% (n = 3) of the medium-sized enterprises, 66.7% (n = 4) of the large enterprises described them as useful. The respondents were also asked about the performance of Fimea. Nearly half (41.2%, n = 14) of the micro enterprises, 46.2% (n = 12) of the small enterprises, 62.5% (n = 5) of the medium-sized enterprises and 50% (n = 3) of the large enterprises were dissatisfied with the performance of Fimea (Table 7).

**Table 7:** ICT systems and performance of Fimea regarding the MDR, n = 74

ICT systems in the MDR (e.g., EUDAMED*, NANDO**) are useful			Agree	Neither agree nor disagree	Disagree
Enterprise size	Micro	n	17	10	7
		%	50%	29.4%	20.6%
	Small	n	14	9	3
		%	53.8%	34.6%	11.5%
	Medium-sized	n	3	3	2
		%	37.5%	37.5%	25%
	Large	n	4	2	0
		%	66.7%	33.3%	0%
The regulatory authority (Fimea) has performed its duties well in the MDR***					
Enterprise size	Micro	n	10	10	14
		%	29.4%	29.4%	41.2%
	Small	n	6	8	12
		%	23.1%	30.8%	46.2%
	Medium-sized	n	1	2	5
		%	12.5%	25%	62.5%
	Large	n	1	2	3
		%	16.7%	33.3%	50%

EUDAMED = European Database on Medical Devices.

**NANDO = New Approach Notified and Designated Organisations.

***MDR = Medical Device Regulation.

Note: Percentages may not total 100% due to rounding or multiple responses.

The respondents were asked about the role of health technology in health care. The results show that it is regarded as important among the enterprises. All (100%, n = 6) of the respondents from the large enterprises stated that health technology improves the effectiveness of health care, enhances the patient and customer experience and improves the quality of care, while 83.3% (n = 5) of the respondents from the large enterprises stated that the utilisation of health technology reduces healthcare costs. Regarding the above, micro, small and large enterprises were fairly unanimous while medium-sized enterprises tended to be more doubtful (Table 8).

**Table 8:** The role of health technology in health care, n = 74

Health technology improves the effectiveness of health care			Agree	Neither agree nor disagree	Disagree
Enterprise size	Micro	n	31	2	1
		%	91.2%	5.9%	2.9%
	Small	n	25	1	0
		%	96.2%	3.8%	0%
	Medium-sized	n	5	2	1
		%	62.5%	25%	12.5% 0
	Large	n	6	0	
		%	100%	0%	0%
**Health technology enhances the patient and customer experience**					
Enterprise size	Micro	n	30	3	1
		%	88.2%	8.8%	2.9%
	Small	n	23	3	0
		%	88.5%	11.5%	0%
	Medium-sized	n	4	3	1
		%	50%	37.5%	12.5%
	Large	n	6	0	0
		%	100%	0%	0%
**The utilisation of health technology reduces healthcare costs**					
Enterprise size	Micro	n	31	2	1
		%	91.2%	5.9%	2.9%
	Small	n	20	3	3
		%	76.9%	11.5%	11.5%
	Medium-sized	n	4	3	1
		%	50%	37.5%	12.5%
	Large	n	5	1	0
		%	83.3%	16.7%	0%
**Health technology can be used to improve the quality of care**					
Enterprise size	Micro	n	31	2	1
		%	91.2%	5.9%	2.9%
	Small	n	23	2	1
		%	88.5%	7.7%	3.8%
	Medium-sized	n	5	2	1
		%	62.5%	25%	12.5%
	Large	n	6	0	0
		%	100%	0%	0%

Note: Percentages may not total 100% due to rounding or multiple responses.

## Discussion

This study aimed to examine how managers and regulatory professionals in health technology enterprises perceive the application of the MDR and what are their information needs regarding the MDR. This study adds to the understanding of the health technology industry and the role of regulation in the industry. Health technology is one of the most rapidly developing industries in Finland [34] and it has an essential role in Finnish healthcare [35 (p.5)].

When taking a closer look at the medical device classification [3, 16], Class I and IIa devices are the most manufactured medical devices, while only a few of the enterprises manufactured high-risk Class III and Class I subclass (Is, Im, Ir) medical devices. Based on this study, the size of an enterprise affects how many medical devices it manufactures. In practice, larger enterprises tend to have more medical devices on their portfolio.

Information related to the MDR is fragmented and enterprises must search for information from multiple information sources. Fimea supervises the regulatory compliance of medical devices and operators [26] and, based on this study, plays an important role in disseminating information about the MDR. The role of Fimea, EU websites and medical device consultants as an information source is regarded as significant. The respondents stated that information concerning the MDR was available although they had challenges understanding the available information. Knowledge and expertise regarding the MDR were perceived as being at a satisfactory level in the enterprises. However, decision-making based on the available information could be quite challenging. It is interesting to note that most of the respondents were highly educated as most of them had a graduate or doctoral degree.

When taking a closer look at training in the MDR, we note that Fimea plays an important role as a training provider. Also, in-house training in the MDR is widespread. The topics of training have been diverse, ranging from clinical trials to quality management systems. The need for training is also discussed in the Medical Device Coordination Group (MDCG) document, which suggests that existing and new Notified Bodies should offer their staff more training, coaching and internship activities to foster capacity building [50].

Considering the essential role of Fimea as an information and training provider, it is somewhat controversial that a significant number of respondents stated that they were dissatisfied with the performance of Fimea regarding the MDR. Based on our findings, we believe it is important to evaluate the challenges faced by Fimea and how it could improve its performance.

Large enterprises stated that the MDR improves product traceability and patient safety and therefore fulfils the aim of the regulation [1]. Smaller enterprises tended to be more sceptical about this. Micro enterprises in particular did not seem to believe that the MDR guarantees fair market access. Based on this study, the ICT systems [22, 23] in the MDR are still not operating at their full potential. When looking more closely at the role of health technology in health care, micro, small and large enterprises have noted that health technology improves the effectiveness of health care, enhances the patient and customer experience [38] and can be used to improve the quality of care. Health technology can also reduce healthcare costs, which are increasing in Western countries. [32 (p.4)].

At the time of the study, there were approximately 400 health technology enterprises in Finland. Previous studies have ended up with slightly smaller figures. As a reference, Kulvik, Kuusi and Pajarinen [32 (p.80)] analysed 290 Finnish health technology enterprises. In a report by Grönlund et al., [51] a questionnaire was sent out to 364 health technology enterprises and received 35 responses, generating a 9.6% response rate. When evaluating the validity and reliability of this study, it is important to note that the number of respondents (n = 74) did not permit generalization to the whole population and in this case to the medical device industry. Thus, we conducted a descriptive analysis of the data. The findings of this study should be situated within the context of Finnish health technology enterprises. Some of the conclusions are based on respondents’ perceptions. It should also be noted that the questionnaire may have been answered by more than one person from the same enterprise. Another limitation of the study is that due to the distribution of the questionnaire on social media, the questionnaire may also have been answered by a person who did not represent the study’s target group. However, the number of these respondents was small.

When assessing the rather low response rate of our study (16.3%), it must be noted that there are some signs of declining response rates in various fields such as management accounting [52]. Especially, the studies involving top managers and organizational representatives have lower response rates than the studies which focus on non-executive employees [53, 54]. As an example in Cycyota’s and Harrison’s meta-analysis the median response rate of top managers was 32% [55]. In addition, online surveys have a lower response rate when compared with other survey modes [56]. One explanation for the nonresponse in this study could be survey fatigue.

Despite the limited number of respondents, this study provides valid findings that offer important insights into the topic. Health technology enterprises play an important role in health care since these enterprises manufacture and distribute goods and services to satisfy the needs and demands of public health. Future research could focus on the EU’s approach to the regulation of artificial intelligence [57] since it may have similarities to the implementation of the MDR.

## Conclusions

This study shows that health technology enterprises have welcomed the MDR and understand its role in ensuring the safety and transparency of medical devices. Although the respondents are familiar with the MDR, there is a partial gap in information and its use. The available information does not properly fit the information needs of users. Thus, the conclusion is that there is a need to improve information quality. The respondents would probably like to have access to a platform with centralised information related to the MDR. To some extent, the MDR is regarded as a burden for smaller enterprises. Such outcomes are quite logical as larger enterprises have more resources at their disposal than smaller enterprises. The respondents were not very familiar with the ICT systems provided by the EU and based on the findings of this study, it is important to highlight the benefits of the ICT systems and to develop them to better meet the needs of enterprises.

## Data Availability

The datasets are not publicly available due to privacy restrictions of the participants but are available from the corresponding author on reasonable request.
